# Technique for Orthodontic Clinical Photographs Using a Smartphone

**DOI:** 10.1155/2022/2811684

**Published:** 2022-01-22

**Authors:** Al Imran Shahrul, Nabilla Shukor, Noraina Hafizan Norman

**Affiliations:** ^1^Centre for Family Oral Health, Faculty of Dentistry, Universiti Kebangsaan Malaysia, Kuala Lumpur 50300, Malaysia; ^2^Centre of Paediatric Dentistry and Orthodontic Studies, Faculty of Dentistry, Universiti Teknologi MARA, Sungai Buloh 4700, Selangor, Malaysia

## Abstract

Smartphone cameras have advanced at a rapid rate. With this advancement, it is possible to take high quality orthodontic clinical photographs using a smartphone. The aim of this article is to describe the technique to take orthodontic clinical photographs using a smartphone.

## 1. Introduction

Within the field of orthodontics, high-quality clinical photographs are essential as a diagnostic tool, to monitor treatment progress, and for educational and medicolegal purposes. Orthodontic clinical photographs consist of extraoral and intraoral photographs [1]. Orthodontic clinical photographs may be taken by a professional medical photographer, orthodontist, or dental assistant [2].

Smartphones have become one of the most essential pieces of equipment in our daily lives. The latest smartphones are packed with features that make them much more than devices for making calls. In orthodontics, smartphones are known to be used for remote monitoring of treatment progress, cephalometric analysis, and taking orthodontic clinical photographs [3–7].

**Figure 1:**
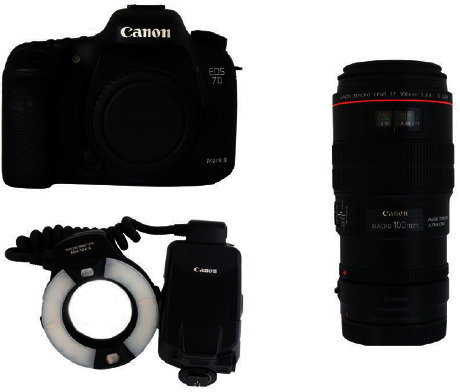
DSLR camera, macro lens and ring flash.

The current gold standard equipment for taking orthodontic clinical photographs is a DSLR (digital single-lens reflex) camera with a 100 mm macro lens and macro ring flash [8, 9] (Figure 1). The high cost and large size of a DSLR camera and its lenses might deter certain dentists from using this equipment, and they might instead opt to use their smartphone camera [10]. Compared to a DSLR camera, a smartphone has the advantages of being smaller, lighter, cheaper, more user-friendly, and able to perform more tasks. Taking personal photographs using a smartphone is relatively simple, but capturing high-quality orthodontic clinical photographs is harder. To produce acceptable orthodontic photographs that meet the standards of clinical records requires the dentist to have the proper equipment and use proper techniques.

The purpose of this article is to explain and illustrate the technique of taking orthodontic clinical photographs using a smartphone.

## 2. Techniques

### 2.1. Camera Settings

#### 2.1.1. General Settings

**Figure 2:**
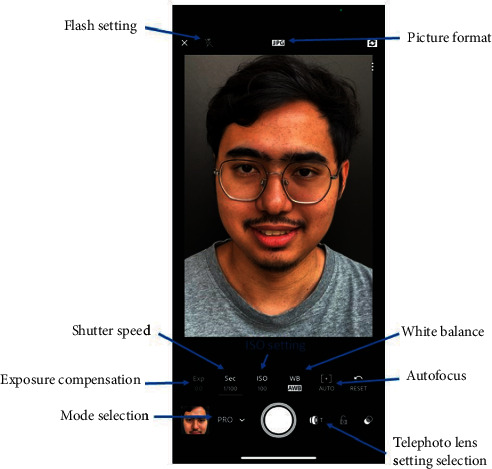
Example of manual settings.

In the preinstalled camera application (app), select the manual shooting mode. If the smartphone does not have a manual mode, a third-party app such as Adobe Lightroom Mobile can be installed which allows the camera to use manual mode (Figure 2). The dentist should select the JPEG format picture quality if it is available. The larger RAW file type should only be selected if the dentist wishes to edit the photo. In the settings, the white balance setting should be set to Auto. The camera will then select the best white balance for the photo depending on the lighting conditions.

The author uses autofocus to achieve focus. While the settings provide an option to use manual focus, in the author's experience, the current manual focus feature in a smartphone is not as accurate as autofocus, increasing the chances of out-of-focus photographs. The focusing point for extraoral photos should be the eye, and for intraoral photos, the canine tooth. Finally, the exposure compensation should be set to zero.

#### 2.1.2. Shutter Speed, Aperture, and ISO

**Figure 3:**
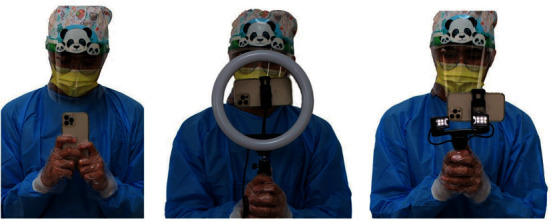
Methods of holding a smartphone.

The author recommends a shutter speed faster than 1/100 second and suggests two methods to reduce the occurrence of camera shake, which may produce a blurred image (Figure 3). First, hold the smartphone with both hands as close to your body as possible. Next, tuck in your arms. This will turn your body into a stable platform like a tripod. The author finds this method to be effective in reducing camera shake.

Select the highest aperture value possible in the camera. A higher aperture number increases the depth of field. Most smartphones have shorter focal lengths. The shorter focal length results in the operator being closer to the patient, thereby reducing the depth of field. Hence, a higher aperture setting is required in smartphones.

The ISO should be set at the lowest value available in the camera. Once all the camera settings have been made, the dentist can preview the exposure of the photograph. If the exposure is deemed too dark, the author suggests that the dentist first increase the light intensity of the continuous light. If the maximum intensity has been reached and the photograph is still underexposed, only then should the dentist increase the ISO value. Increasing the ISO is the last method to increase the exposure, because increasing its value will produce a noisier photograph.

### 2.2. Patient Positioning

The extraoral view is taken either in the standing or seated position [1, 11]. As a reference, the author uses the patient's interpupillary plane for the frontal view and the Frankfort plane in the profile and three-quarter view [12]. The plane should be parallel to the floor for every view. The intraoral view is taken in the dental chair. The patient is seated in a 45-degree angle for the anterior and buccal views, whereas the occlusal picture is taken in the supine position.

Modern smartphones are equipped with multiple lenses with different focal lengths. The author recommends that the dentist select the telephoto lens with the greatest focal length. Once the telephoto lens is selected, the author walks closer to the patient until the patient's image fills the frame. It is best to avoid zooming, if possible, as zooming will significantly reduce the quality of the photograph. If the camera is too close to the patient, only then will the author use the zoom feature of the camera until an acceptable distance from the patient is achieved.

### 2.3. Environment, Background, Lighting, and Accessory Equipment

The environment where the photograph is taken plays a huge role in the quality of the image produced [13]. Extraoral photographs are best taken in a well-lit room with many windows and a daylight colour temperature light (5000 K–6500 K) [14]. The brighter the environment, the less additional external lighting is required.

A plain, nondistracting white or black background is typically used in orthodontic clinical photography [1]. Because of the limitations of mobile dental photography, the author does not recommend a white background due to the presence of shadows, especially in profile or three-quarter view. Instead, a darker background should be used to hide shadows.

**Figure 4:**
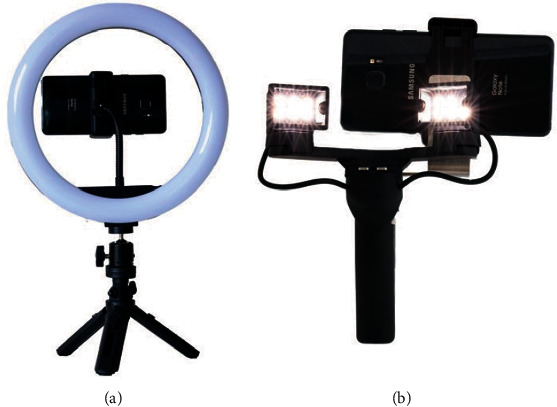
LED ring light (a) and intraoral LED light (b).

One of the trickiest components of mobile dental photography is lighting. One method to overcome this shortcoming is to use a continuous light source [14]. The author recommends an affordable portable LED ring or box light (Figure 4). For intraoral photographs, the author uses a dedicated LED light to illuminate the teeth.

**Figure 5:**
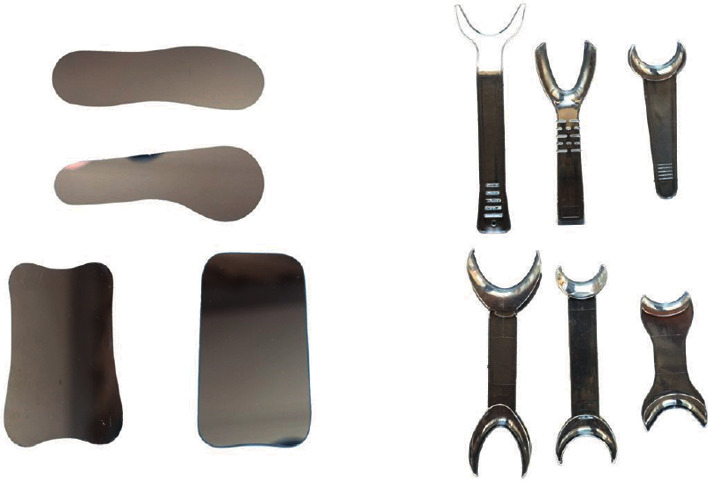
Intraoral mirrors and cheek retractors.

Accessory equipment such as a cheek retractor and mirror is essential for intraoral photography (Figure 5). Various sizes of retractors and mirrors are available on the market. The size should be selected according to the patient. A transparent retractor and a mirror with a handle are recommended.

## 3. Discussion

**Figure 6:**
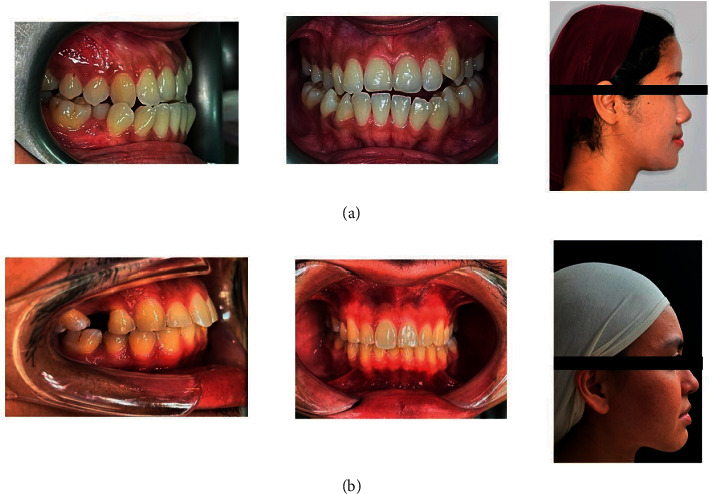
Photos taken (a) without the proper technique and (b) with the proper technique.

In the hands of an experienced dentist, the picture quality of a DSLR camera system is far superior to that of a smartphone. Furthermore, there are certain technical differences between taking a picture with a smartphone and a DSLR. These differences are summarised in Table 1. Hence, for a dentist who uses a DSLR daily, there is a need to get refamiliarized with the new settings. However, the inferior picture quality of smartphones does not mean that photographs taken with a smartphone are unacceptable for an orthodontic clinical record. Based on the author's experience, any modern-day smartphone, from the top-of-the-line to an average model, can capture quality orthodontic photos with the proper technique (Figure 6). The author strongly believes that a photo taken with a smartphone is better than no photo at all.

The author would like to emphasize the importance of data privacy when taking clinical photos with a smartphone [15]. Due to the connectivity of smartphones to the internet, the possibility of the picture being leaked online is significantly higher. The author recommends that clinical photos should not be kept together with personal photos. Using a separate phone or folder for clinical photos is best. To ensure that unauthorized personnel do not gain access to the photos, the folder should be password protected. Sharing of photos via messenger or social media is prohibited without the consent of the patient. The connectivity of smartphones does provide an advantage. Photos stored on the phone can be transferred immediately to a computer or cloud storage. This ensures that, in the unfortunate event of a lost phone or SD card failure, there is a backup file available. Finally, when a smartphone is no longer being used, its memory should be erased.

**Figure 7:**
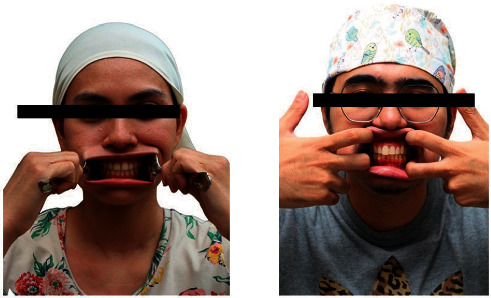
Retraction of the cheek using a spoon and fingers.

Smartphone photography not only benefits the dentist but also the patient [16]. With the current COVID-19 pandemic, more dentists have incorporated teledentistry in their daily practice [17]. The consultation can occur via live video feed or by the patient sharing their clinical photographs. The patient is unlikely to have a dedicated camera system with a macro lens and flash to provide the necessary photographs. The only tool at their disposal is probably their smartphone. The techniques explained previously can be used at home with a few modifications. For instance, the author asks the patient to replace the cheek retractor with a spoon or their fingers and the intraoral mirror with a makeup mirror, if available (Figure 7). If there is no continuous LED lighting, only then does the author recommend that the patient use the smartphone camera flash. The settings recommended to the patient will highly depend on the patient's knowledge of technology. If the dentist feels the patient is not well-versed in smartphone photography, more automatic settings will be recommended.

## 4. Conclusions

Orthodontic clinical photographs can be taken with a smartphone by using the correct methods. The dentist should ensure that the photographs are taken in the optimum environment with the optimum lighting and using the recommended settings.

## Figures and Tables

**Table 1:** Summary of the technical differences between a DSLR and a smartphone in taking orthodontic photographs.

	DSLR	Smartphone
Picture format	JPEG/RAW	JPEG/RAW
Lens focal length	100 mm focal length lens with macrocapabilities	Depends on the smartphone manufacturer (select the longest focal length lens)
Additional lighting	A macro ring or twin flash attached to the camera hot shoe	External continuous LED light
Aperture	Extraoral: F8 Intraoral: F22	Depends on the smartphone manufacturer (select the highest aperture number possible)
ISO	100	100 or lowest possible
Shutter speed	1/100 per second	1/100 per second
White balance	Neutral	Auto
Autofocus settings	Manual focus	Autofocus

## Data Availability

No data were used to support this study.

## References

[1] Sandler J., Murray A. (2010). Clinical photography in an orthodontic practice environment part 1. *Orthodontic Update*.

[2] Sandler J., Dwyer J., Kokich V. (2009). Quality of clinical photographs taken by orthodontists, professional photographers, and orthodontic auxiliaries. *American Journal of Orthodontics and Dentofacial Orthopedics*.

[3] Mohan A., Sivakumar A., Nalabothu P. (2021). Evaluation of accuracy and reliability of Oneceph digital cephalometric analysis in comparison with manual cephalometric analysis—a cross-sectional study. *BDJ Open*.

[4] Dalessandri D., Sangalli L., Tonni I. (2021). Attitude towards telemonitoring in orthodontists and orthodontic patients. *Dentistry Journal*.

[5] Klaus K., Stummer A.-L., Ruf S. (2021). Accuracy of a smartphone application measuring snoring in adults—how smart is it actually?. *International Journal of Environmental Research and Public Health*.

[6] Vaid N. R., Hansa I., Bichu Y. (2020). Smartphone applications used in orthodontics: a scoping review of scholarly literature. *Journal of the World Federation of Orthodontists*.

[7] Gupta G., Vaid N. R. (2017). The world of orthodontic apps. *APOS Trends in Orthodontics*.

[8] Marcato L., Sandler J. (2018). The best choice of equipment to obtain high quality standardised results in intra-oral photography-a comparison between the common practice in the UK and the gold standard set by the literature. *Journal of Visual Communication in Medicine*.

[9] Shahrul A. I. (2021). Mirrorless cameras in orthodontic practice. *Journal of Orthodontics*.

[10] Chan N., Charette J., Dumestre D. O., Fraulin F. O. (2016). Should “smart phones” be used for patient photography?. *Plastic Surgery*.

[11] McKeown H. F., Murray A. M., Sandler P. J. (2005). How to avoid common errors in clinical photography. *Journal of Orthodontics*.

[12] Capon T. (2016). Standardised anatomical alignment of the head in a clinical photography studio. A comparison between the frankfort horizontal and the natural head position. *Journal of Visual Communication in Medicine*.

[13] Ahmad I. (2009). Digital dental photography. Part 7: extra-oral set-ups. *British Dental Journal*.

[14] Ahmad I. (2009). Digital dental photography. Part 5: lighting. *British Dental Journal*.

[15] Payne K. F., Tahim A., Goodson A. M., Delaney M., Fan K. (2012). A review of current clinical photography guidelines in relation to smartphone publishing of medical images. *Journal of Visual Communication in Medicine*.

[16] Bianco A., Dalessandri D., Oliva B. (2021). COVID-19 and orthodontics: an approach for monitoring patients at home. *The Open Dentistry Journal*.

[17] Saccomanno S., Quinzi V., Sarhan S., Laganà D., Marzo G. (2020). Perspectives of tele-orthodontics in the COVID-19 emergency and as a future tool in daily practice. *European Journal of Paediatric Dentistry*.

